# Monitoring chaos at the cot-side

**DOI:** 10.1038/s41390-024-03151-1

**Published:** 2024-03-20

**Authors:** Michael J. Beacom, Martin G. Frasch, Christopher A. Lear, Alistair J. Gunn

**Affiliations:** 1 Department of Physiology, The University of Auckland, Auckland, New Zealand; 2 Department of Obstetrics and Gynecology and Institute on Human Development and Disability, University of Washington School of Medicine, Seattle, WA USA

Intraventricular hemorrhage (IVH) affects ~10–20% of preterm infants born before 30 weeks of gestation.^1^ Severe IVH is associated with an increased risk of death and life-long neurodevelopmental disorders such as cerebral palsy^2^ and cognitive, behavioral, and learning difficulties.^3^ Early detection is a major challenge as more than half of IVH cases are clinically silent, and although some cases present with seizures, many have relatively subtle symptoms.^4^ Being able to identify fetuses or neonates at risk of and determine the temporal profile of IVH in real time would be important to understand the windows for prevention or therapeutic intervention.

Scahill et al. in this issue investigated whether sample entropy (SampEn), a non-linear domain measure of the irregularity in heart rate variability (HRV), could predict intraventricular hemorrhage (IVH) and mortality risk in preterm infants within the first 24 h of life.^5^ In a cohort of 389 preterm infants, a total of 29 developed IVH grade 3 or 4, and 31 infants died. Using the open-source PhysioNet cardiovascular toolbox and a logistic regression prediction model to predict links between SampEn, IVH, and mortality, they found a significant correlation with eventual mortality as early as 4 h of life and with severe IVH by 24 h. At 96 h, when it performed best, the combination of SE with the clinical model yielded an area under the curve (AUC) of 0.9 and above for all three outcome groups and performed better than the clinical model alone.

This study examined the pragmatic discriminative value of SampEn but did not seek to understand the physiological meaning behind the measure. SampEn, first introduced by Richman and Moorman (2000), is a metric of entropy in HR signals.^6^ Entropy reflects the level of irregularity and complexity based on the uncertainty within a signal. Technically, SampEn helps to quantify the conditional probability that two sequences of *m* points in an epoch of length *N* remain similar when one consecutive point (*m* + *1)* is added within a tolerance of *r*.^7^ Complexity is related to, but distinct from the variability measured by other (linear) HRV measures of the time and frequency domains.^8^ In the context of cardiovascular regulation, the complexity of HR times series is postulated to reflect the capability of the cardiovascular system to adapt to transient stressors and demands of an ever-changing environment.^9^

It is important to appreciate that the patterns measured by SampEn are often not easily observable to the human eye. Thus, there are two possible interpretations of the patterns observed in this study. First, that breakdown of the physiological inputs that drive HR may lead to unphysiologically regular patterns, as observed in the first ~4–8 h of life (low SampEn) or uncorrelated, irregular random signals, as observed between 24 and 96 h (high SampEn). These two grades of outcomes, one regular and the other random, are actually indicators of low complexity compared to signals taken from a healthy system.^10^ Paradoxically, high SampEn may also imply greater complexity, as complex systems inherently exhibit some irregularity. In the future, alternative non-linear measures like detrended fluctuation analysis (DFA) or newer entropy measures (e.g., fuzzy entropy, entropy rate or other measures that are time-scale-sensitive or probe different embedding dimensions) might offer better differentiation of these features.^11,12^ Nevertheless, these encouraging results strongly suggest that the evolution of FHRV over time can help detect IVH. Indeed, an important question for future studies would be whether the early signature of SampEn suppression extends into the intrapartum or even antenatal periods.

Other potential practical limitations should be considered. SampEn is easily affected by signal artefacts (noise, outliers), which can lead to significant variations in entropy values.^7^ While the authors addressed noise concerns, SampEn is highly parameter-dependent, and incorrect or inconsistent parameter selection can lead to significant changes in the resulting calculated entropy. Pincus and colleagues suggested the standardization of r within the range of 0.1 to 0.25 times the standard deviation of the time-series signal.^13^ Subsequent studies suggest that the r range cannot be easily generalized across datasets.^14,15^ An inappropriate choice of *r* can lead to the so-called ‘flip-flop’ effect, where results differ dramatically and can be statistical unstable, due to its inherent dependence on the Heaviside function. This two-state signal classifier dichotomizes signal vectors into either “dissimilar” or “similar” without any intermediary states.^14^ Further, the present study employed linear regression to prevent overfitting.^5^ While this is a valid approach, it assumes a linear relationship between predictors and HRV, which is not always true for complex physiological systems like HRV.

These observations suggest that future studies should include additional measures as well as SampEn to help predict IVH. More recent methods of measuring entropy have tried to address the technical limitations of SampEn, including so called Fuzzy entropy, Permutation entropy, Distribution entropy and Bubble entropy.^11,12^ Although they sound esoteric, all are readily available through open-source toolboxes.^16^ Alternatively, there is growing evidence that HR patterns measured by the highly comparative time series analysis (HCTSA)^17^ can predict sepsis^18^ and cerebral palsy^19^ in preterm and term neonatal populations; this approach includes multiple time, frequency, and non-linear domain measures.

Overall, this study indicates great potential of open-source software to measure HRV as a screening tool for detecting and tracking the evolution of IVH. Further studies are now needed to better understand the optimal approach.

## References

[1] Dol, J. et al. Timing of neonatal mortality and severe morbidity during the postnatal period: a systematic review. *JBI Evid. Synth.* **21**, 98–199 (2023). 36300916 10.11124/JBIES-21-00479 PMC9794155

[2] Kitai, Y., Hirai, S., Ohmura, K., Ogura, K. & Arai, H. Cerebellar injury in preterm children with cerebral palsy after intraventricular hemorrhage: Prevalence and relationship to functional outcomes. *Brain Dev.* **37**, 758–763 (2015). 25571998 10.1016/j.braindev.2014.12.009

[3] Legge, N., Lutz, T., Wocadlo, C. & Rieger, I. Long-term neurodevelopmental outcome in preterm infants with intraventricular haemorrhage. *J. Paediatr. Child Health* **58**, 1797–1802 (2022). 35837759 10.1111/jpc.16108 PMC9796685

[4] Alkalay, A. L., Galvis, S., Ferry, D. A., Simmons, C. F. & Krueger, R. C. Jr. Hemodynamic changes in anemic premature infants: are we allowing the hematocrits to fall too low? *Pediatrics* **112**, 838–845 (2003). 14523175 10.1542/peds.112.4.838

[5] Scahill, M. D. et al. Sample entropy correlates with intraventricular hemorrhage and mortality in premature infants early in life. *Pediatr. Res*. 10.1038/s41390-024-03075-w (2024). 10.1038/s41390-024-03075-w 38365874

[6] Richman, J. S. & Moorman, J. R. Physiological time-series analysis using approximate entropy and sample entropy. *Am. J. Physiol. Heart Circ. Physiol.* **278**, H2039–H2049 (2000). 10843903 10.1152/ajpheart.2000.278.6.H2039

[7] Lake, D. E., Richman, J. S., Griffin, M. P. & Moorman, J. R. Sample entropy analysis of neonatal heart rate variability. *Am. J. Physiol. Regul. Integr. Comp. Physiol.* **283**, R789–R797 (2002). 12185014 10.1152/ajpregu.00069.2002

[8] Beckers, F., Verheyden, B. & Aubert, A. E. Aging and nonlinear heart rate control in a healthy population. *J. Physiol. Regul. Integr. Comp. Physiol.* **290**, H2560–H2570 (2006). 10.1152/ajpheart.00903.2005 16373585

[9] Francesco, B. et al. Linear and nonlinear heart rate variability indexes in clinical practice. *Comput. Math. Methods Med.* **2012**, 219080 (2012). 22400047 10.1155/2012/219080 PMC3287009

[10] Goldberger, A. L. Fractal variability versus pathologic periodicity: complexity loss and stereotypy in disease. *Perspect. Biol. Med.* **40**, 543–561 (1997). 9269744 10.1353/pbm.1997.0063

[11] Roux, S. G., Garnier, N. B., Abry, P., Gold, N. & Frasch, M. G. Distance to healthy metabolic and cardiovascular dynamics from fetal heart rate scale-dependent features in pregnant sheep model of human labor predicts the evolution of acidemia and cardiovascular decompensation. *Front. Pediatr.* **9**, 660476 (2021). 34414140 10.3389/fped.2021.660476 PMC8369259

[12] Frasch, M. G. et al. Nonlinear properties of vagal and sympathetic modulations of heart rate variability in ovine fetus near term. *Am. J. Physiol. Regul. Integr. Comp. Physiol.* **296**, R702–R707 (2009). 19109371 10.1152/ajpregu.90474.2008

[13] Bošković, A., Lončar-Turukalo, T., Japundžić-Žigon, N. & Bajić, D. The flip-flop effect in entropy estimation. In *Proc. International Symposium on Intelligent Systems and Informatics*, pp 227–230.

[14] Castiglioni, P. & Rienzo, M. D. How the threshold “r” influences approximate entropy analysis of heart-rate variability. *Comput. Cardiol*. **35**, 561–564 (2008).

[15] Lu, S., Chen, X., Kanters, J. K., Solomon, I. C. & Chon, K. H. Automatic selection of the threshold value R for approximate entropy. *IEEE Trans. Biomed. Eng.* **55**, 1966–1972 (2008). 18632359 10.1109/TBME.2008.919870

[16] Flood, M. W. & Grimm, B. EntropyHub: an open-source toolkit for entropic time series analysis. *PLoS ONE* **16**, e0259448 (2021). 34735497 10.1371/journal.pone.0259448 PMC8568273

[17] Fulcher, B. D. & Jones, N. S. hctsa: a computational framework for automated time-series phenotyping using massive feature extraction. *Cell Syst.* **5**, 527–531.e523 (2017). 29102608 10.1016/j.cels.2017.10.001

[18] Niestroy, J. C. et al. Discovery of signatures of fatal neonatal illness in vital signs using highly comparative time-series analysis. *NPJ Digit. Med.* **5**, 6 (2022). 35039624 10.1038/s41746-021-00551-z PMC8764068

[19] Letzkus, L. et al. Heart rate patterns predicting cerebral palsy in preterm infants. *Pediatr. Res*. 10.1038/s41390-023-02853-2 (2023). 10.1038/s41390-023-02853-2 PMC12100623 37891365

